# Mitotherapy for Fatty Liver by Intravenous Administration of Exogenous Mitochondria in Male Mice

**DOI:** 10.3389/fphar.2017.00241

**Published:** 2017-05-09

**Authors:** Ailing Fu, Xianxun Shi, Huajing Zhang, Bin Fu

**Affiliations:** School of Pharmaceutical Sciences, Southwest UniversityChongqing, China

**Keywords:** mitochondrial dysfunction, mitotherapy, fatty liver, lipid accumulation, oxidation injury

## Abstract

Mitochondrial dysfunction is a major and common mechanism in developing non-alcoholic fatty liver disease (NAFLD). Replacement of dysfunctional mitochondria by functional exogenous mitochondria may attenuate intrahepatic excessive lipid and recover hepatocyte function. However, no data shows that mitochondria can be systemically administrated to animals to date. Here we suggest that mitochondria isolated from hepatoma cells are used as a mitotherapy agent to treat mouse fatty liver induced by high-fat diets. When the mitochondria were intravenously injected into the mice, serum aminotransferase activity and cholesterol level decreased in a dose-dependent manner. Also, the mitotherapy reduced lipid accumulation and oxidation injury of the fatty liver mice, improved energy production, and consequently restored hepatocyte function. The mitotherapy strategy offers a new potential therapeutic approach for treating NAFLD.

## Introduction

Non-alcoholic fatty liver disease (NAFLD) is the most common form of chronic liver disease that affects a high proportion of the population. Patients with NAFLD are at high risk of developing steatohepatitis with fibrosis and cirrhosis. Experimental and clinical evidence have suggested that mitochondrial dysfunction is a major reason of developing NAFLD, since mitochondria are important determinants of cellular lipid metabolism and oxidant stress (Serviddio et al., 2010; Auger et al., 2015). Recent studies indicate that improvement of mitochondrial function by physical exercise and/or antioxidants prevents fat accumulation in patients with NAFLD (Guo et al., 2015; Salomone et al., 2016). However, mitochondrial DNA and/or proteins are always irreversibly damaged in NAFLD, thus exercise and antioxidants provide limited hepatocyte protection (Di Maso and Bellentani, 2009; Qu and Zhang, 2010; Sun et al., 2016).

Replacement of dysfunctional mitochondria by functional mitochondria may be the most direct approach for treating NAFLD. Studies have suggested that isolated mitochondria can enter cultured mammalian cells by simple co-incubation (Kitani et al., 2014a; Kesner et al., 2016), then rescue receiving cells against cell injury induced by mitochondrial deficiency (Katrangi et al., 2007; Islam et al., 2012). However, no data shows that exogenous mitochondria can be systemically administrated into animals as a mitotherapy agent for treating mitochondrion-associated diseases to date.

Non-alcoholic fatty liver disease develops as a result of high-fat diets, and the disease may progress in a manner involving increased lipid accumulation and production of oxidants. In this study, we prepared fatty liver mice that were fed with high-fat diets for 8 weeks, and identified that the mitochondria isolated from cultured human hepatoma cell (HepG2) could be systemically injected into the mice, then reduce lipid accumulation and excessive oxidants, and consequently restore hepatocyte function in the mice. The result for the first time suggests that the mitotherapy strategy will be a novel therapeutic approach for treating NAFLD and potential for other mitochondrion-associated diseases.

## Materials and Methods

### Animals

Healthy male C57BL/6J mice (SCXK [Jing 2006-2009]), weighing 18 ∼ 22 g, were used in the study. The mice were purchased from Chongqing Medical University, Chongqing, China. Animals were maintained under standard housing conditions with *ad libitum* access to standard laboratory mouse chow and water. All animal experiments were carried out in accordance with guidelines evaluated and approved by the Animal Committee of Southwest University, China.

### Lentiviral Vector Transfection

**FIGURE 1:**
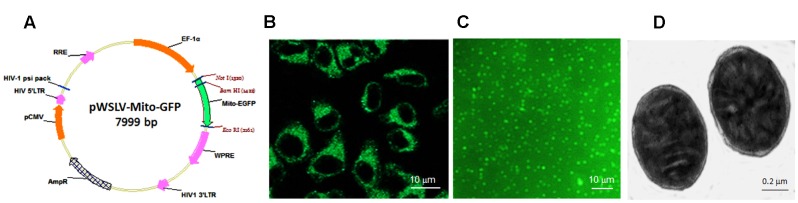
**Lentiviral vector transfection and isolated mitochondria. (A)** Lentiviral vector plasmid pWSLV/Mito-GFP. **(B)** The vector was expressed in the cytosol of HepG2 cells. **(C)** The isolated mitochondria showed green fluorescence. **(D)** Mitochondrial structure under TEM.

The plasmid pCMV/Mito/GFP (Invitrogen, Cambridge, MA, USA), encoding a fusion protein of green fluorescence protein (GFP) and mitochondrial targeting sequence from subunit VIII of human cytochrome c oxidase, was cleaved by NotI and EcoRI. Then the Mito-GFP gene fragment was cloned into the lentiviral vector plasmid (pWSLV; Beijing Weishang Lide Biotech. Co., Beijing, China) to produce pWSLV/Mito-GFP (**Figure 1A**). Meanwhile, human embryonic kidney 293T cells were cultured in 6-cm culture dishes in DMEM medium supplemented with 10% fetal bovine serum (FBS; Gibco, USA) (Wu et al., 2015). When cell confluence reached 80%, pWSLV/Mito-GFP were transfected into the cells by using Lipofectamine 3000 (Invitrogen, Cambridge, MA, USA). The expression of fusion protein Mito-GFP was observed by using a confocal microscope (Zeiss LSM 510, Germany) after 24 h transfection. Vectors were harvested 2 days post-transfection and filtered through a 0.45-μm pore size filter (Rochester, NY, USA). The vectors were then further concentrated by ultracentrifugation (Beckman Coulter, CA) at 4°C and resuspended in PBS. Subsequently, the lentiviral vectors were added into HepG2 cell media (Wu et al., 2016), and green fluorescence was observed under the confocal microscope. Then fluorescence-activated cell sorting (FACs) was used to sort florescence positive cells with flow cytometry (BD FACSVantage, CA). The positive cells were further cultured to obtain the stable cell line with GFP-tagged mitochondria.

### Isolation of Mitochondria

HepG2 cells were digested by 0.25% trypsin/EDTA at 37°C for 5 min, and then washed twice by PBS. Mitochondrial isolation was performed according to the manufacturer’s protocol of mitochondrial isolation and purification kit (Pierce, WI, USA). Briefly, the cells were homogenized in cold Isolation Buffer, and then the homogenate was centrifuged at 800 *g* for 5 min at 0 ∼ 4°C. Subsequently, the supernatant was collected and resuspended in Isolation Buffer for another centrifugation at 10 000 g for 10 min. The mitochondrial precipitate was washed with Isolation Buffer for two times and placed at 4°C before use. The mitochondria were, respectively, observed under a florescence microscope (Olympus, Japan) and a transmission electron microscope (TEM; Hitachi, Japan). Mitochondrial concentration was determined by BCA assay.

### *In Vivo* Distribution Analysis

Saline solution of the GFP-tagged mitochondria (0.2 ml) and GPP control (stored in our laboratory) (Qian et al., 2015), were respectively, injected slowly via tail veins into mice at a dose of 0.5 mg/kg body weight. Two hours later, the mice were deeply anesthetized with 4% chloral hydrate and transcardically perfused with PBS (0.01 M, pH 7.4) to remove blood. Mouse brain, lung, liver, kidney and muscle were, respectively, excised and placed in cassette of In Vivo Imaging System FX Pro (Carestream Health Inc., Rochester, NY, USA) for fluorescence imaging. The images were captured with X-ray and fluorescence, and overlaid according to the protocol of the manufacturer.

Subsequently, the tissues were fixed by 4% paraformaldehyde in PBS, and dehydrated with 10, 20, 30% sucrose successively. Frozen sections (30 μm) were cut with a cryomicrotome (Leica, Germany), and the section fluorescence was observed under the confocal microscope.

### Preparation of Fatty Liver Model and Mitochondrial Treatment

C57BL/6J mice were randomly assigned to four group (*n* = 12 for each group). The mice in first group fed with a standard chow diet (fat content ∼4%) were served as normal control, and the mice in other three groups received intragastric administration of a high-lard-fat and high-cholesterol diet that was composed of 87.3% standard diet, 10% lard, 2% cholesterol, 0.2% propylthiouracil, 0.5% sodium cholate daily (Park et al., 2016; Bae et al., 2017), in which sodium cholate can improve gastrointestinal absorbance of fat, and propylthiouracil can increase fat synthesis and decrease fat decomposing through inhibiting the function of thyroxine. The fat content in standard mouse chow diet is about 4% (%weight of the total), while the total fat content in the high-fat diet, including 87.3% standard diet and 10% lard (99.6% fat content in lard), is about 13.4% (i.e., 87.3% × 0.04 + 9.96 = 13.4%; %weight of the total). Eight weeks later, two mice removed randomly from each group were euthanized to evaluate liver damage. For mitotherapy group 1, the mice were intravenously injected mitochondria (0.5 mg/kg body weight) once in three days for three times; for mitotherapy group 2, the mice received mitochondria for six times. For fatty liver group, the mice were intravenously administrated equal volume of saline. Following mitochondrial treatment, all mice were fasted for 12 h, and then euthanized by overdose pentobarbital sodium. Mouse serum and liver tissues were separated for tissue examination and biochemical measurements.

### Tissue Examination

Liver tissue was fixed in 4% paraformaldehyde, then successively dehydrated with 10, 20, 30% sucrose. The tissues were embedded in paraffin and cut into 30 μm thin slices on the cryomicrotome. The frozen sections were stained with Oil Red O to detect lipid deposit (Zhou et al., 2017), and images were taken by an optical microscope (Olympus, Japan).

Moreover, ultrathin liver sections were taken for mitochondrial observation under a transmission electron microscope (TEM; H7500, Hitachi, Japan) according to the operation manual (Electron microscope center of Chongqing Medical University, Chongqing, China). Briefly, the liver tissue was fixed in polyformaldehyde/glutaraldehyde solution (2%/2.5%) and kept at 4°C. Sections (60 nm thick) were cut and loaded onto copper grids, and then were stained with uranyl acetate for 10 min. Pictures were taken under TEM at an accelerating voltage of 160 kV.

### Biochemical Assay

After mitochondrial injection, mice in the four groups were, respectively, euthanized. Blood was collected by cardiac puncture for serum preparation. Activity of serum alanine aminotransferase (ALT) and aspartate aminotransferase (AST), and content of cholesterol (TC) and low density lipoprotein-cholesterol (LDL-C) were, respectively, determined using an automatic biochemistry analyzer (labcompare, USA).

Moreover, the livers were quickly excised and snap-frozen in liquid nitrogen for preparation of tissue homogenate. Intrahepatic triglyceride (TG) and TC contents were measured by the automatic biochemistry analyzer. Levels of ATP, reactive oxygen species (ROS), glutathione (GSH), malondialdehyde (MDA), and mitochondrial cytochrome oxidase and superoxide dismutase (SOD) activity in liver homogenates were, respectively, determined by using commercial kits (Nanjing Jiancheng Biotech. Ltd. Co., Nanjing, China), where ATP level was assayed by luciferase-fluorescein bioluminescent method (Sevin et al., 1988), ROS was detected by Fenton reaction and Griess reagent (Lin et al., 2014), GSH was determined by the spectrophotometric method based on the use of Ellman’s reagent (Buege and Aust, 1978), the MDA level was assayed by monitoring thiobarbituric acid reactive substance formation (Beutler, 1975; Fu et al., 2006), mitochondrial cytochrome oxidase and SOD activity were measured by using resazurin method and pyrogallol autoxidation method, respectively (Marklund and Marklund, 1974; Wang et al., 2015).

### Statistical Analysis

Data were expressed as mean ± SEM Results were analyzed by one-way analysis of variance (ANOVA) followed by Tukey post hoc test. Differences were considered significant when *p* < 0.05 (denoted by ^∗^ or ^#^), and highly significantly when *p* < 0.01 (denoted by ^∗∗^ or ^##^).

## Results

### Morphology of Isolated Mitochondria

After lentiviral vector transfection for 48 h, the cytosol of HepG2 cells showed strong green fluorescence (**Figure 1B**). Then GFP-tagged mitochondria were isolated from the cells. Under microscope, the mitochondria displayed spherical shape with good dispersion (**Figure 1C**). Also, double membrane structure and cristae of the isolated mitochondria remained intact, observing by TEM (**Figure 1D**).

### Mitochondrial Distribution in Mice

**FIGURE 2:**
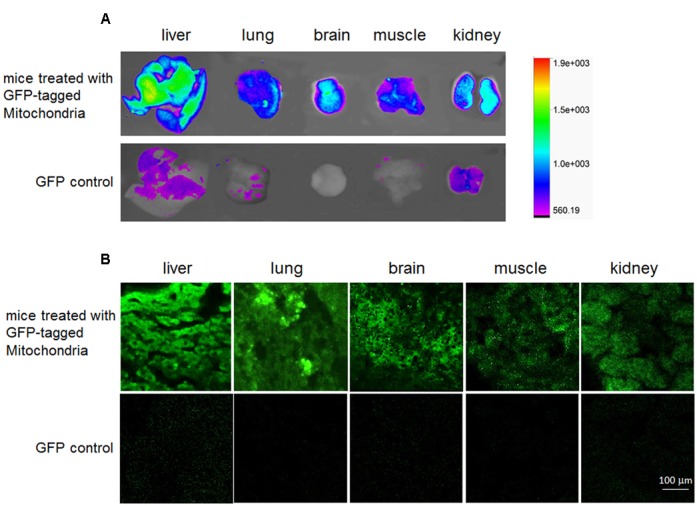
**Distribution *in vivo* of the GFP-tagged mitochondria after intravenous administration.** Tissue fluorescence was evaluated by tissue imaging **(A)** and tissue sections **(B)**.

To determine the *in vivo* distribution of exogenous mitochondria after intravenous injection, the GFP-tagged mitochondria were used. After mice were injected with the mitochondria (0.5 mg/kg) for 2 h, obvious fluorescence appeared in mouse liver, lung, brain, muscle, and kidney, while week fluorescence exhibited in liver, kidney, and lung following GFP injection (**Figure 2A**), indicating that the GFP-tagged mitochondria could enter mouse tissue cells. Also, the imaging of frozen sections further identified that the exogenous mitochondria could enter various tissue cells after systemic injection (**Figure 2B**).

### Reduction of Serum Transaminase and Lipid after Mitochondrial Treatment

**FIGURE 3:**
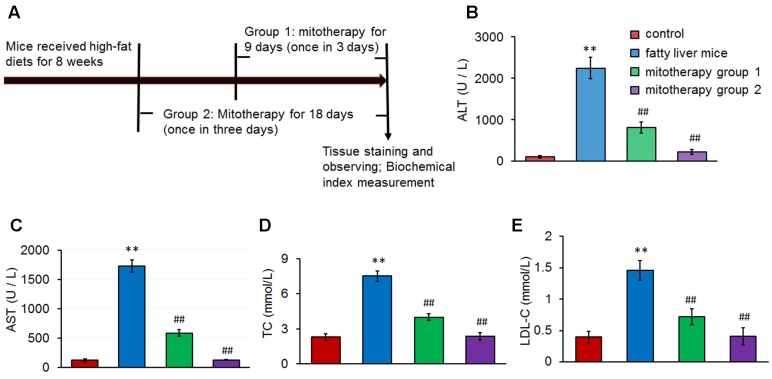
**Serum transaminase and lipid levels in the mice treated with high-fat diets and mitochondria. (A)** Experimental protocol of mitotherapy for mouse fatty liver induced by high-fat diets. **(B)** ALT activity. **(C)** AST activity. **(D)** TC level. **(E)** LDL-C level. Data were expressed as mean ± SEM (*n* = 10 mice for each group). ^∗∗^*p* < 0.01 compared with the control; ^##^*p* < 0.01 compared with the fatty liver mice.

After the fatty liver mice received 0.5 mg/kg body weight of mitochondrial injection once in three days for three times or six times, sera were collected for transaminase activity and lipid content measurement (**Figure 3A**). The results showed that serum ALT and AST levels significantly increased in the fatty liver mice (**Figures 3B,C**), suggesting that the high-lard-fat and high-cholesterol diets induced cell injury in mice, which was consistent with previous data (Han et al., 2015; Langhi et al., 2017). However, after mitochondrial administration for 3 times (mitotherapy group 1), transaminase activities decreased, from 2246.38 ± 256.30 (ALT) and 1729.78 ± 105.60 U/L (AST), to 816.97 ± 128.34 (ALT) and 585.21 ± 56.42 U/L (AST), respectively (**Figures 3B,C**). Moreover, transaminase activities nearly recovered to the control levels after mitochondrial administration for six times (mitotherapy group 2), displaying a dose-dependent manner in mitotherapy for mouse fatty liver.

In addition, serum TC and LDL-C levels increased after the mice were fed with the high-fat diets (*p* < 0.01) (**Figures 3D,E**). However, the levels of TC and LDL-C decreased significantly in the mitotherapy group 1 compared with the fatty liver mice (*p* < 0.01), and recovered to the control levels in mitotherapy group 2 (**Figures 3D,E**), suggesting that exogenous mitochondria reduced cell injury and improved serum lipid metabolism.

### Treatment of Mouse Fatty Liver with Exogenous Mitochondria

**FIGURE 4:**
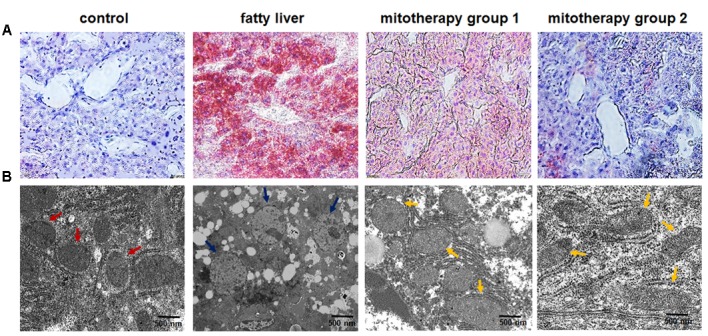
**Pathological changes of the liver tissues of the mice treated with high-fat diets and mitochondria. (A)** Liver tissues were stained with Oil Red O. The excessive accumulation of lipid droplets in mouse liver appeared after the mice were fed with high-fat diet. Mice in mitotherapy group 1 were intravenously injected mitochondria three times in 9 days (once in 3 days), while mice in mitotherapy group 2 received mitochondria six times in 18 days. Representative photomicrographs (200×). **(B)** Ultrathin sections for mitochondrial observation under TEM. The red arrows pointed to normal mitochondria, while the blue ones pointed to the swelling mitochondria, and the yellow ones pointed to the restored mitochondria.

Oil red O staining showed an obvious fat deposit in the mouse liver after the mice were fed with the high-fat diets for 8 weeks (**Figure 4A**), while the lipid droplets decreased after mitochondrial injection for three times, and almost disappeared after six times’ mitochondrial administration (**Figure 4A**). Also, TEM analysis of liver sections revealed that mitochondria showed obvious swelling, and the cristae disappeared in the fatty liver mice, suggesting that the mitochondrial structure and function were damaged by the high-fat diets (**Figure 4B**). However, the mitochondrial morphology was improved after three times’ mitochondrial injection, and exhibited almost normal structure after 6 times’ administration (**Figure 4B**).

**FIGURE 5:**
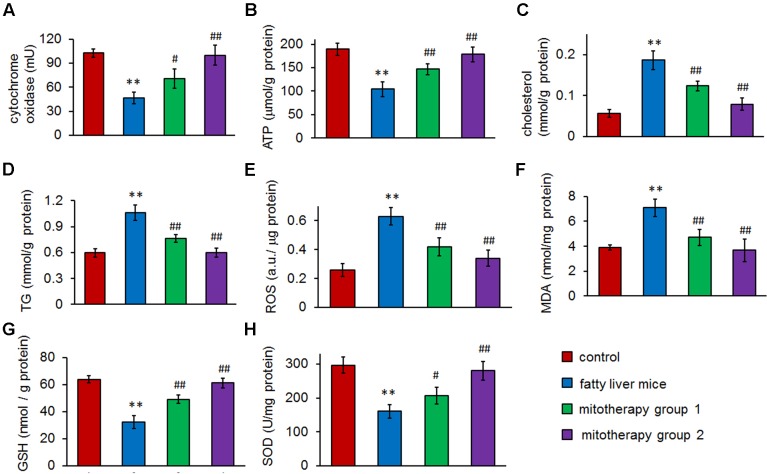
**Therapeutic effects of mitochondria on high-fat diet-induced fatty liver. (A)** Mitochondrial cytochrome oxidase activity. **(B)** ATP content. **(C)** Hepatic cholesterol. **(D)** Hepatic TG. **(E)** ROS content. **(F)** MDA level. **(G)** GSH level. **(H)** SOD activity. Data were expressed as mean ± SEM (*n* = 10 mice for each group). ^∗∗^*p* < 0.01 compared with the control; ^#^*p* < 0.05, ^##^*p* < 0.01 compared with the fatty liver mice.

The results of biochemical measurement showed that mitochondrial cytochrome oxidase activity was significantly reduced in the fatty liver mice (*p* < 0.01) (**Figure 5A**), which was consisted with the TEM observing of mitochondrial injury. However, the activity remarkably increased in the mice of mitotherapy group 1, and nearly reached control activity in the mitotherapy group 2, from 46.64 ± 7.24 mU to 70.43 ± 12.00 mU (group 1) and 99.94 ± 12.44 mU (group 2), respectively (**Figure 5A**). Accordingly, ATP content of liver homogenate in fatty liver mice significantly decreased compared with the control mice (**Figure 5B**), while three times’ mitotherapy caused a 40.6% increase in ATP level, from 104.33 ± 15.62 μmol/g protein in the fatty liver mice, to 146.75 ± 12.14 μmol/g protein in the mitotherapy group 1 (**Figure 5B**). Also, no significant difference showed in the ATP level between the control group and mitotherapy group 2 (**Figure 5B**). Additionally, the levels of cholesterol and TG decreased after mitochondrial administration (**Figures 5C,D**). These results indicated that exogenous mitochondria could restore hepatocyte mitochondrial function.

To determine the effects of mitochondria on the high-fat diet-induced oxidative injury, levels of ROS, MDA, GSH, and activity of SOD in mouse liver homogenates were, respectively, measured. The results showed that after the mice were fed with high-fat diets for 8 weeks, the hepatic ROS and MDA levels increased, and cellular antioxidant GSH (the first line of defense against oxidative injury in the form of ROS) content and SOD activity remarkably decreased (**Figures 5E–H**). However, after mitochondrial administration, both ROS and MDA (the lipid peroxidation product) significantly reduced compared with the fatty liver mice, while GSH content and SOD activity increased accordingly (*p* < 0.01). Also, there was no obvious difference of ROS, MDA, GSH, and SOD levels between normal control group and the mitotherapy group 2 (**Figures 5E–H**). These results suggested that the mitotherapy could rescue the hepatocytes from oxidative injury.

## Discussion

Mitochondria are the main organelles devoted to lipid oxidation and ATP production. Thus, mitochondrial dysfunction will induce excessive lipid deposition and absence of energy in cells. Although the number of patients with NAFLD has gradually increased in proportion with the obesity, there are no established approaches for treating NAFLD. Here we showed that exogenous mitochondria isolated from HepG2 cells could restore hepatocyte function in high-fat diet-induced fatty liver mice. The study will provide a novel therapeutic strategy for NAFLD and potentially for other types of mitochondrion-deficient diseases.

Studies have suggested that exogenous mitochondria can directly transform into cultured xenogeneic cells and improve their mitochondrial function. For example, mitochondria isolated from human uterine endometrial gland derived mesenchymal cells (EMCs) enter H9c2 cardiomyoblasts by simple co-incubation, and rescue the cells’ mitochondrial respiratory function (Kitani et al., 2014b). Another example is that Katrangi et al. (2007) transfer murine mitochondria into human rho0 cells and identify that the mitochondria can improve respiratory function. Moreover, heterologous mitochondria can improve embryonic development in fertilized murine zygotes after microinjection, suggested by Pinkert et al. (1997). Nevertheless, no data shows that the exogenous mitochondria can be systemically administrated to animals. Here we used genetically GFP-tagged mitochondria that were isolated from the cells transfected with lentiviral vector, and determined *in vivo* distribution and function of exogenous mitochondria after systemic injection. The results revealed that all mice treated with the mitochondria survived and no abnormality appeared, and the mitochondria entered various tissue cells, including liver, lung, brain, muscle and kidney. The widespread distribution of administered mitochondria *in vivo* will be benefit for a group of multisystemic mitochondrial diseases.

Hepatocytes are enriched in mitochondria, and each cell contains about 800 mitochondria. Proper mitochondrial function is critical for the maintenance of the cell life. Accumulating evidence have indicated that hepatic mitochondrial dysfunction play a critical role in development and pathogenesis of NAFLD (Grattagliano et al., 2012), and Rector et al. (2010) also showed that mitochondrial dysfunction precedes hepatic steatosis in the natural history of NAFLD, although the mechanisms underlying this dysfunction are still unclear. One of the possible reasons might be that high-fat diet causes mitochondrial stress in liver, inducing impairment of mitochondrial structure and function (Lee, 2015). Afterward, defective mitochondrial β-oxidation and respiratory chain increase lipid toxic metabolites, which contributes to the pathogenesis of NAFLD.

Improvement of mitochondrial function will prevent NAFLD through promoting β-oxidation and reducing lipid accumulation. For example, restored mitochondrial function by LY2405319 can treat NAFLD by enhancing fatty acid oxidation and decreasing oxidative stress and lipid peroxidation in the liver (Lee et al., 2016). Our results also suggested that supplement of functional exogenous mitochondria reduced hepatic fat accumulation in a dose-dependent manner.

Oxidative stress is considered an important factor in producing hepatocyte injury and inflammation reaction associated with NAFLD (Paradies et al., 2014). Excessive ROS enhance the peroxidation of fatty acids in mitochondrial membranes to produce lipid peroxidation productions, such as MDA (Barrera et al., 2016). In fatty liver mice, MDA remarkably increased, which indicated that the cells were damaged by overproduction of ROS. Also, low levels of GSH and decreased expression and/or activity of antioxidant enzymes have additional deleterious effects on fatty liver, whose mechanisms have been identified by substantial studies *in vitro* and *in vivo* (Ali et al., 2016; Yang et al., 2016).

Mitochondria are key players in cellular redox metabolism (Kalinina et al., 2008). Functional mitochondria contains various enzymatic and non-enzymatic antioxidant defense systems to protect the organelles from oxidative damage, such as GSH and multiple GSH-linked antioxidant enzymes, SOD and ascorbate-GSH cycles (Lismont et al., 2015). Therefore, functional mitochondria could diminish the deposited fat without overproduction of ROS. In this study, supplement of functional mitochondria increased GSH level and SOD activity, and reduced ROS and MDA content in the liver, which was considered as therapeutic mechanisms of functional mitochondria against oxidant injury.

## Conclusion

The present study is the first to demonstrate a therapeutic effect of exogenous mitochondria against high-fat diet-induced fatty liver in mice. The data showed that the mitochondria could directly target tissue cells after intravenous injection. The novel approach of mitotherapy decreased lipid content and restored cellular redox balance, consequently attenuated lipid deposit and prevented cell injury. Findings from the current study offer new potential therapeutic avenues for treatment of NAFLD and potentially other mitochondrion-associated liver diseases.

## Ethics Statement

This study was carried out in accordance with the recommendations of Animal Use and Safety Regulation, Animal Committee of Southwest University, Chongqing, China. The protocol was approved by the Animal Committee of Southwest University, Chongqing, China.

## Author Contributions

AF prepared the animal model and wrote the manuscript. XS isolated the mitochondria and stained the sections. XS and HZ carried out the biochemical index measurement. BF did the cell culture and vector transfection. All authors have read and approved the final manuscript.

## Conflict of Interest Statement

The authors declare that the research was conducted in the absence of any commercial or financial relationships that could be construed as a potential conflict of interest. The reviewers PM, EN and handling Editor declared their shared affiliation, and the handling Editor states that the process nevertheless met the standards of a fair and objective review
